# Hepatoid Adenocarcinoma of Stomach: Emphasis on the Clinical Relationship with Alpha-Fetoprotein-Positive Gastric Cancer

**DOI:** 10.1155/2019/6710428

**Published:** 2019-12-13

**Authors:** Er-Bao Chen, Yi-Chou Wei, Hai-Ning Liu, Cheng Tang, Meng-Ling Liu, Ke Peng, Tianshu Liu

**Affiliations:** ^1^Department of Medical Oncology, Zhongshan Hospital, Fudan University, Shanghai, China; ^2^Department of Gastroenterology, Zhongshan Hospital of Fudan University, Shanghai, China; ^3^Center of Evidence-Based Medicine, Fudan University, Shanghai, China

## Abstract

**Aims:**

Both hepatoid adenocarcinoma of stomach (HAS) and alpha-fetoprotein-positive gastric cancer (AFPGC) are rare but aggressive subtypes of gastric cancer, but few studies focus on the clinicopathologic differences and prognostic factors between them because of their rarity and histologic overlap. And the significance of AFP level in HAS prognosis was not well studied.

**Methods:**

41 patients with AFPGC and 52 patients with HAS were included in this study. The clinicopathologic features were compared by Chi-square analysis. Prognostic factors for overall survival (OS) and disease-free survival (DFS) were analyzed with the Kaplan-Meier method.

**Results:**

The patients with HAS were of a younger age compared with AFPGC, and nearly 60% of tumor located in the gastric antrum and the gastric fundus of cardia. The OS of AFPGC was shorter than that of HAS, due to a higher rate of metastasis. Furthermore, the survival analysis showed that HAS with high AFP expression (AFP^High^ HAS) had a significantly poorer OS compared to HAS with low AFP expression (AFP^Low^ HAS) (*P*=0.046).

**Conclusions:**

Compared with AFPGC, the patients of HAS were of a younger age and had less rate of liver and other organ metastasis. The serum AFP level was a sensitive prognostic indicator for OS. Therefore, much attention should be paid to AFP^High^ HAS in clinical practice.

## 1. Introduction

Alpha-fetoprotein (AFP), a kind of oncogenic glycoprotein, is originally found in the human fetus and is mainly synthesized and secreted by the fetal liver, yolk sac, and some gastrointestinal cells, which rapidly decreases after birth [1, 2]. In clinical practice, a rise of serum AFP is a well-known suitable serum tumor biomarker used to screen or monitor hepatocellular carcinoma (HCC), tumors of gonadal origin, or yolk sac tumors [3–5]. However, elevated serum AFP has also been observed in multiple cancers of various other organs [6, 7], and gastric cancers were the most common among these tumors [8–10]. In 1970, Bourreille's group first described a case of gastric adenocarcinoma with liver metastasis, and its serum and pathological specimen were positive for AFP, which led to the term “AFP-positive gastric cancer (AFPGC)” [11]. Since then, AFPGC has been reported all over the world but mostly in Asia, with an estimated incidence of 2.3%–7.1% of all gastric cancers [12]. APFGC has a higher incidence of venous invasion and liver metastasis compared with AFP negative gastric cancer [13, 14].

As a diagnostic rule, serum AFP levels of most AFPGC were only slightly higher than normal, but in some cases, they were as high as several thousand ng/mL or more, even beyond the detection limit [15–17]. Some researchers observed that certain lesions mimicked HCC-like morphology under the light microscope, especially in these AFPGCs with high serum level of AFP. The cells were composed of large, polygonal cells with abundant eosinophilic cytoplasm. In 1986, Ishikura et al. [18] proposed a new term “hepatoid adenocarcinoma” to describe a type of histological feature mimicked HCC. Hepatoid adenocarcinoma (HAC) is a malignant cancer manifesting outside of liver that presents morphological areas identical to that of HCC. HAC is defined by the minimum criteria of characteristic hepatoid area regardless of its proportion within the tumor mass or AFP production in WHO classification [19]. HAC also arises in multiple organs, such as stomach (63%), ovary (10%), lung (5%), gallbladder (4%), uterus (4%), and pancreas (4%) [20]. Compared with other types of gastric cancer, hepatoid adenocarcinoma of stomach (HAS) progresses rapidly and metastasizes to lymph node or liver. The risk of recurrence in HAS patients is high, even after radical resection [21]. Many studies demonstrated that both AFPGC and HAS had more aggressive biobehavior and were usually associated with poor prognosis due to advanced stage, easy recurrence, and frequent liver and lymph node metastasis [8, 14].

Although the histologic features of APFGC and HAS have been studied by pathologists, their clinical features and prognostic factors have not been well studied. In this retrospective study, to clarify the clinical features and prognostic factors between AFPGC and HAS, we investigated the histologic and clinicopathologic features of AFPGC and HAS and clarified the difference between AFPGC and HAS. Furthermore, we first divided the HAS into two subgroups according to the serum AFP 100 *μ*g/L. We found that HAS patients with high AFP expression had a worse overall survival compared to those with low AFP expression.

## 2. Materials and Methods

### 2.1. Patients and Data Collection

HAS was diagnosed based on the World Health Organization system. Primary gastric cancer exhibiting a typical hepatoid component was diagnosed as HAS [22, 23]. The diagnoses of AFPGC were identified on primary gastric cancer with elevated AFP in the serum without hepatoid differentiation in the tissue. 41 patients with AFPGC and 52 patients with HAS were found in Zhongshan Hospital, Fudan University, between 2010 and 2017. Tumor stage was classified according to the 7^th^ edition of the American Joint Committee on Cancer (AJCC). Data were retrieved from patients' medical charts, and follow-up data were acquired by telephone and the clinical database. Written informed consent had been obtained, and this study was approved by the hospital ethics committee.

### 2.2. Immunohistochemistry

All patients were diagnosed based on surgical specimens or gastroscopy biopsy specimen. Hematoxylin and eosin (HE) staining sections retrieved from HAS and AFPGC groups were reviewed by two gastrointestinal pathologists. The fixed tissue specimens were processed to be embedded in paraffin and subsequently sectioned for HE and immunohistochemical staining. The pathologic diagnosis of HAS was based on histologic characteristics similar to HCC. Histologically, there was no quantitative requirement for diagnosis of hepatic differentiation, and partial cases of hepatic differentiation foci can also be diagnosed. Ten slides of every specimen were identified to find the hepatic differentiation. According to HE staining, the specimens were diagnosed into histological types: hepatoid adenocarcinoma and normal adenocarcinoma.

### 2.3. Statistical Analysis

The clinicopathologic variables between HAS and AFPGC were estimated by Chi-square and Fisher's exact test. The Kaplan–Meier method was employed for calculating overall survival and disease-free survival rates, and log-rank test was used for comparison between survival curves. The level of statistically significance was *P* values <0.05. All calculations were performed with the SPSS (version 23.0) statistical package (IBM Corporation, Armonk, NY, USA).

## 3. Results

### 3.1. Histologic Features of HAS

**Figure 1:**
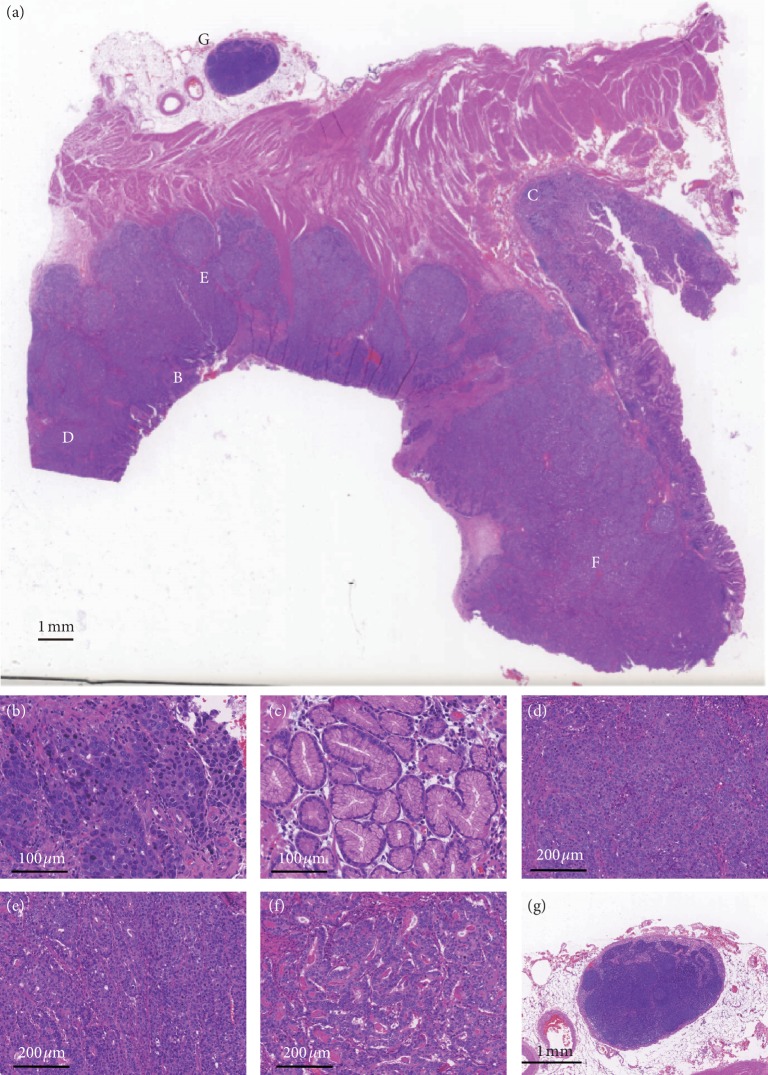
Histologic features of hepatoid adenocarcinoma of stomach (HAS). (a) Hepatoid component (lower part of image) and normal gastric mucosa component (right part of image) are visible. (b) Tumor cells with abundant eosinophilic granular hepatocyte-like neoplastic cells and nucleus were large and ovoid and contained 1-2 nucleoli. (c) Normal gastric mucosa morphology. (d) Typical hepatoid area of HAS proliferation in solid nest fashion. (e) Typical hepatoid area of HAS proliferation in trabecular pattern. (f) Focally, the HAS tumor cells form glandular structures. (g) The lymph node that has not been invaded by the tumor.

The primary lesions consisted of normal component and hepatoid component (Figure 1(a)).

The tumor cells of HAS were cuboidal or polygonal with abundant eosinophilic granular hepatocyte-like neoplastic cells, and the nucleus was large and ovoid and contained 1–2 nucleoli (Figure 1(b)). The normal component of HAS exhibited normal gastric mucosa (Figure 1(c)). In the typical areas of hepatoid component, tumor cells tended to be arranged in solid nests (Figure 1(d)) or trabecular fashion (Figure 1(e)). Some proportions of the tumors exhibited distinct glandular differentiation (Figure 1(f)). In this specimen, the lymph node had not been invaded by the tumor (Figure 1(g)).

### 3.2. General Characteristics of HAS and AFPGC

The clinical and pathological characteristics of patients with AFPGC or HAS are summarized in Table 1. Overall, a total of 41 cases of HAS and 52 cases of AFPGC were included in this study. Of HAS group, there were 30 male (73.2%) and 11 female (26.8%) patients (male-to-female ratio = 2.73 : 1). The median age was 66 years (range: 43–82). The most common initial presentation was abdominal pain (15/41, 36.6%) and others including hematochezia, hematemesis, choking, back pain, chest congestion, and weakness; of note, 13 patients (13/41, 31.7%) were occasionally found in physical examination. Of AFPGC group, there were 39 male (75%) and 13 female (25%) patients (male-to-female ratio = 3 : 1). The median age was 65 years (range: 26–76). The most common initial presentation was abdominal distension (17/52, 32.7%) and others including abdominal pain and hematemesis. It is worth noting that incidental finding had occupied a certain proportion of HAS (13/41, 31.7%) and AFPGC (8/52, 15.4%). These suggested that the chance of incidental finding was twice as much as in HAS as in AFPGC. Regarding tumor location, HAS mainly occurs in the gastric antrum (12/41, 29.3%) and the gastric fundus of cardia (12/41, 29.3%), whereas AFPGC mainly occurred in the gastric body (19/52, 36.5%) and the gastric fundus of cardia (16/52, 30.8%). The AFP level in the serum of 23 patients was more than 100 ng/mL in HAS, while the serum AFP of 30 patients with AFPGC was higher than 100 ng/mL. Patients with APFGC had a significantly higher incidence of liver metastasis than those with HAS.

### 3.3. Comparison of Clinicopathological Features between AFPGC and HAS

30 HAS patients and 16 AFPGC patients received surgical treatment (two patients with neoadjuvant treatment were excluded). The clinicopathological features of 30 HAS and 16 AFPGC patients were summarized in Table 2. Both groups were predominantly male (73.33%, 75%, respectively). More than half patients diagnosed with HAS were under 60 years; nearly half of patients diagnosed with AFPGC were under 60 years. Most HAS tumors (21/30, 70%) were less than 5 cm in diameter, while nearly half of APFGCs were less 5 cm in diameter. For Laruen's classification, 14 cases (14/30, 46.67%) of HAS were identified as intestinal type, and only 6 cases of AFPGC were identified as intestinal type. One-third (11/30, 36.67%) of HAS occurred in the stomach antrum, and one-third of AFPGC occurred in the stomach body. 22 cases were poorly differentiated (22/30, 73.33%) in the HAS and 12 cases displayed poor differentiation (12/16, 75%). The tumors in the HAS infiltrating depth of T1-T2 and T3-T4 were 6 and 24. There were 22 cases of lymph node metastasis, 15 cases of vascular invasion, and 9 cases of nerve invasion in the patients with AFPGC. The tumors in the patients with AFPGC infiltrating depth of T1-T2 and T3-T4 were 1 and 15, respectively. There were 13 cases of lymph node metastasis, 11 cases of vascular invasion, and 10 cases of nerve invasion in the patients with HAS (Table 2).

### 3.4. Liver Metastasis and Other Organ Metastases

Of AFPGC group, the overall incidence of liver metastasis was 55.8% (29/52), including 48.1% (25/52) synchronous and 7.7% (4/52) metachronous liver metastasis. 17 (17/52, 32.7%) patients with AFPGC underwent surgical treatment, 9 cases of radical total gastrectomy, 4 cases of radical distal gastrectomy, 3 cases of radical proximal gastrectomy, and 1 case of neoadjuvant treatment. During the last follow-up, 4 patients had liver metastasis and 6 patients had other organ metastasis including peritoneum and pancreas. 35 (35/52, 67.3%) patients did not undergo surgery treatment. 25 (25/35, 71.4%) cases were diagnosed with synchronous liver metastasis, and 10 (10/35, 28.6%) cases were diagnosed with other organ metastasis at the time of presentation.

Of HAS group, the overall incidence of liver metastasis was 26.8% (11/41), including 17.1% (7/41) synchronous and 9.8% (4/41) metachronous liver metastasis. 31 (31/41, 75.6%) patients with HAS underwent surgical treatment, 12 cases of radical total gastrectomy, 14 cases of radical distal gastrectomy, 4 cases of radical proximal gastrectomy, and 1 case of neoadjuvant treatment. During the follow-up, 4 patients had liver metastasis and 6 patients had other types of organ metastasis including peritoneum, pancreas, lung, bone, and pelvic. 10 (10/41, 24.4%) patients did not undergo surgical treatment, 7 (7/10, 70%) cases were diagnosed with liver metastasis at the time of presentation, and 3 (3/10, 30%) cases were diagnosed with other organ metastasis. PVTT is also frequently reported as a prognostic factor for liver cancer, with 2 cases in HAS and 3 in AFPGC. Of note, the difference was not statistically significant regarding PVTT in these two groups.

### 3.5. Analysis of Prognosis Difference between HAS and AFPGC by Surgery Specimens

**Figure 2:**
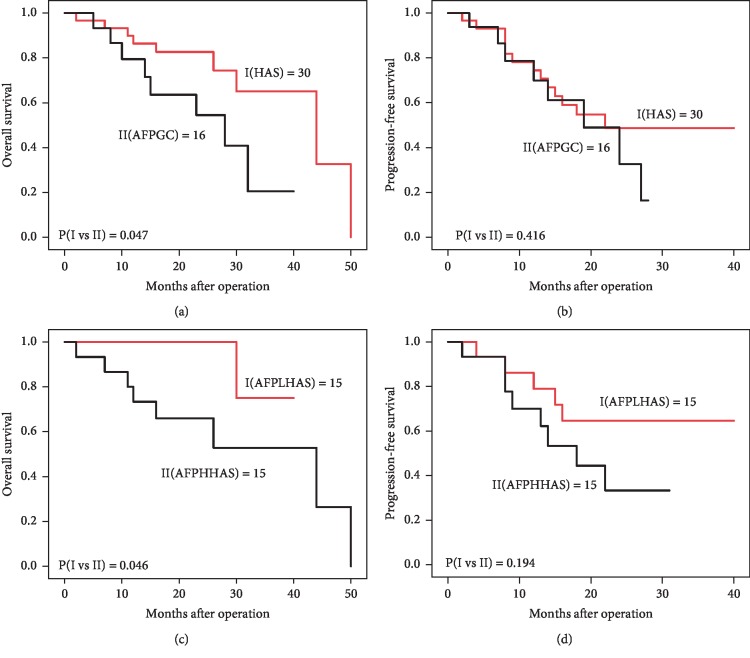
Kaplan–Meier plots of overall survival and disease-free survival of hepatoid adenocarcinoma (HAS) and alpha-fetoprotein-positive gastric cancer (AFPGC). Overall survival (a) outcomes were significantly worse in patients with AFPGC than in patients with HAS. Disease-free survival (b) outcomes revealed no significant difference between patients with HAS and patients with AFPGC. (c, d) Based on a cutoff value of 100 ng/mL of AFP in the HAS, there was statistically significant survival difference in OS (c), but there was no statistically significant survival difference in DFS (d).

The overall survival time (OS) of the AFPGC patients after surgery was 5–40 months (mean 15.5 months), and 3-year cumulative survival rate of the 16 patients was 6.25% (1/16). The disease-free survival time (DFS) of the AFPGC patients after surgery was 4–27 months (mean 13.5 months). The OS of HAS patients after surgery was 7–50 months (mean 20 months), and 3-year cumulative survival rate of 30 patients was 10% (3/30). The DFS of HAS patients after surgery was 2–40 months (mean 16 months). Previous literatures reported that the prognosis of the patients with AFPGC was better than that of patients with HAS. Surprisingly, we found that the OS of the patients with AFPGC was worse than that of patients of HAS (Figure 2, *P*=0.047), but the difference was not statistically significant regarding DFS in these two groups. Considering that slightly high level of AFP may not be necessary to produce an immune-suppressive microenvironment, HAS samples were dichotomized to low AFP expression group (AFP^Low^ HAS, *n* = 15) and high group (AFP^High^ HAS, *n* = 15), with 100 mg/mL AFP level serving as the cutoff value to further investigate the clinical significance of AFP level in HAS. Then, the differences of the general data and the clinicopathological features are shown in Table 3. There was no statistical difference between the two groups in multiple clinical parameters, including sex, tumor location, tumor size, tumor differentiation, Lauren type, vascular invasion, nerve invasion, and liver metastasis. Surprisingly, we observed that the AFP^High^ HAS groups exhibited a younger age compared to the AFP^Low^ HAS (*P*=0.008).

## 4. Discussion

There are quite different definitions about AFPGC. Some studies defined “AFPGC” as “gastric cancer with AFP higher than normal in the serum,” which was the definition Boureille used in the beginning. Later, some researchers define “AFPGC” as “immunohistochemical AFP positivity of tissues.” In 1981, Kodama et al. verified the expression of AFP in gastric cancer, through immunohistochemistry, and established the concept of “AFP-producing gastric cancer” [24] and some other researchers defined “AFPGC” as “positive of AFP in both serum and tissue” [17]. Histological types of AFPGC can be classified as gastric hepatoid, enteroblastic, papillotubular adenocarcinoma, and yolk sac tumors [13]. These concepts had many intersections and separate sections. Our study defined “AFPGC” as “serologic AFP positive and histologically without hepatoid differentiated gastric cancer”. In 1985, Ishikura et al. defined the concept of HAS after reporting an investigation of AFPGC with morphological features mimicking HCC. Subsequent researchers found that some patients with hepatoid adenocarcinoma did not express AFP [25]. However, since a proportion of HAS patients which do not express AFP were reported, Nagai et al. [9] proposed that HAS should be diagnosed based on its histological characteristics, irrespective of its capacity to produce AFP. HAS was considered to represent gastric adenocarcinoma with hepatic differentiation and morphological similarity to hepatic cells. In this study, we used Nagai's definition to HAS.

The clinical presentations of HAS and AFPGC are similar and lack specific clinical symptoms with many symptoms of common gastric cancer such as abdominal pain, gastric distention, back pain, reduced appetite, and hematemesis [26, 27]. Notably, incidental finding had occupied a certain proportion of HAS (13/41, 31.7%) and AFPGC (8/52, 15.4%). High percentage of occult onset may explain the poor prognosis in both HAS and AFPGC. Inagawa et al. investigated 85 HAS patients (mean age, 63.5, range, 44–87 years with a male-to-female ratio of 2.3 : 1) and reported that relatively HAS had occurred more frequently in middle-aged men than in elder men [21]; these findings were consistent with our findings. We found that both HAS and AFPGC have a similar male-to-female rate, but the age of onset of HAS tended to be middle age, whereas that of AFPGC was prone to elder age. Inagawa reported that HAS originated in the gastric antrum in 60% of patients. Only 13% of patients were diagnosed with early-stage HAS. Our data showed that nearly 60% HAS originated in the gastric antrum (12/41, 29.3%) and the gastric fundus of cardia (12/41, 29.3%), whereas AFPGC mainly originated in the gastric body (19/52, 36.5%) and the gastric fundus of cardia (16/52, 30.8%). There was no doubt that the majority of both AFPGC and HAS patients presented with liver, lymph node, and other organ metastases. Surprisingly, we investigated that the rate of liver or other organ metastasis of AFPGC was more than that of HAS, which is contradictory to the previous understanding. The difference may be due to three reasons. (1) Most studies do not strictly distinguish between the two concepts. Liu et al. groups [28] included only HAS with AFP positive, while some HAS with normal AFP were missing. (2) Patients with HAS or AFPGC in our hospital came from all over the country, so regional distribution may be one of the reasons. (3) All research about HAS including our own research had the shortcoming of small sample size. To determine which of the two cancers has the worst prognosis, more investigation with a larger sample size will be required.

As two rare subtypes of gastric cancer, HAS and AFPGC have been reported by many literatures to show more aggressive biobehavior and poorer prognosis than common gastric cancer. However, the prognostic differences between HAS and AFPGC had been controversial. In the current study, we found that AFPGC group presented at a higher incidence of liver metastasis than HAS, which was contrary to previous some studies. Lin et al. reported that the incidence of lymph node and liver metastasis was statistically higher (91.4% versus 60.7%, and 27.6% versus 4.4%). Additionally, we found that the 1-, 2-, and 3-year overall survival rates of APFGC were 75%, 25%, and 6.5%, and 1-, 2-, and 3-year overall survival rates of HAS were 86.6%, 36.7%, and 10%. Chang et al. reported that the 1- and 3-year survival rates of AFPGC were 37.5% and 8.3%. The 3-year survival rates for patients with gastric cancer with 20 ng/mL < AFP < 300 ng/mL and AFP > 300 ng/mL were 28.9% and 7.7% [29]. Our results suggested that AFPGC had more aggressive behavior than HAS. The exact molecular mechanism remains unclear. It was proposed that AFP and other secretory proteins formed immunosuppressive microenvironment and therefore enhanced tumor invasiveness and aggressiveness. However, the role of AFP in HAS or AFPGC prognosis had not be well studied. Inoue M et al. reported that the 5-year survival rate was 34%, and survival after surgery was found to not be linked to the preoperative serum AFP level [30]. However, other studies suggested that a high expression of serum AFP was an independent prognostic indicator [31]. The definition of AFPGC by Inoue et al. was “preoperative AFP level exceeding 40 ng/mL with a decrease after gastrectomy, or raised preoperative AFP level (10–39 ng/mL) and resected tumor showing histologically characteristic features or immunohistochemically positive AFP production [30].” Of note, “histologically characteristic features” in the definition were regarded as “three subtypes: hepatoid type; yolk sac tumor-like type; and fetal gastrointestinal type [10].” And they found that preoperative serum AFP levels showed no correlation with patient prognosis. On the other hand, definition of AFPGC by Chen et al. was “serum AFP-positive patients [31].” Different definitions of AFPGC in two studies led to different results. Inoue et al.'s study included many pathological subtypes. Chen et al.'s study also included two pathological subtypes. It is hard to tell which is the best description of this disease. Thus, more precise criteria for pathological definition are necessary for a better understanding of this pathology subtype, so our study defined “AFPGC” as “serologic AFP positive and histologically without hepatoid differentiated gastric cancer” to compare the role of “AFP” and “hepatoid feature” in the gastric cancer. We found that there was higher frequency of liver metastasis in AFP^High^ HAS than that of AFP^Low^ HAS. Furthermore, we found that AFP^High^ HAS had a significantly poorer OS compared to AFP^Low^ HAS, but these had no significant difference regarding DFS.

## 5. Conclusions

Both AFPGC and HAS have more aggressive behavior characterized by a high frequency of lymph node and liver metastasis and thus led to poorer prognosis than common gastric cancer. The prognosis of HAS is not worse than that of AFPGC. The HAS with high AFP had a younger age and a poorer prognosis compared to HAS with low AFP level. As a result of worse biological behavior, more attention should be paid to HAS with high AFP; further investigation is needed in the future.

## Figures and Tables

**Table 1:** Comparison and clinicopathological characteristics between HAS- and AFP-elevated gastric cancer (AFPGC).

Characteristics	HAS (*n* = 41)	AFPGC (*n* = 52)	*P* value
Age			**<0.001**
≤60	30	18	
>60	11	34	
Sex			0.841
Male	30	39	
Female	11	13	
Initial presentation			**0.049**
Abdominal pain	15	12	
Abdominal distension	8	17	
Medical examination	13	8	
Hematemesis	2	1	
Choked up	1	6	
Others	2	8	
Location			**0.041**
Cardiac stomach bottom	12	16	
Body	4	19	
Antrum	12	11	
Gastric helicobacter	0	1	
Angle	6	2	
Whole stomach	7	2	
Residual stomach	0	1	
Serum AFP			0.877
>100	23	30	
<100	18	22	
Liver metastasis			**0.005**
Yes	11	29	
No	30	23	
Operation			**<0.001**
Yes	31	17	
No	10	35	
Curability			1.000
Curative surgery	30	16	
Neoadjuvant	1	1	
PVTT			1.000
Yes	2	3	
No	39	49	
Other metastasis			0.405
Yes	16	16	
No	25	36	

**Table 2:** Comparison and clinicopathological characteristics between HAS- and AFP-elevated gastric cancer with surgery (AFPGC).

	HAS (*n* = 30)	AFPGC (*n* = 16)	*P* value
Sex			1.000
Male	22	12	
Female	8	4	
Age			0.292
≤60	18	7	
>60	12	9	
Tumor size			0.082
≤5	21	7	
>5	9	9	
Lauren			0.550
Intestinal	14	6	
Nonintestinal	16	10	
Location			0.065
Cardiac stomach bottom	8	4	
Body	2	7	
Antrum	11	1	
Angle	4	1	
Whole stomach	5	2	
Gastric helicobacter	0	1	
Differentiation			1.000
Well differentiated	8	4	
Poorly differentiated	22	12	
*T*			0.576
*T*1	2	1	
*T*2	4	0	
*T*3	19	10	
*T*4	5	5	
*N*			0.605
*N*0	8	3	
*N*1	7	2	
*N*2	7	4	
*N*3	8	7	
Cancer nodules			0.709
0	20	11	
1-2	8	3	
3 and above	2	2	
Vascular invasion			0.222
Yes	15	11	
No	15	5	
Nerve invasion			**0.033**
Yes	9	10	
No	21	6	
Surgery			0.292
Subtotal	18	7	
Total	12	9	
Liver metastasis			0.558
Yes	4	4	
No	26	12	
Other metastasis			0.350
Yes	6	6	
No	24	10	

**Table 3:** Comparison and clinicopathological characteristics between AFP-low and AFP-high HAS.

	AFP^High^ HAS (*n* = 15)	AFP^Low^ HAS (*n* = 15)	*P* value
Sex			0.682
Male	12	10	
Female	3	5	
Age			**0.008**
≤60	13	5	
>60	2	10	
Tumor size			0.427
≤5	12	9	
>5	3	6	
Lauren			0.715
Intestinal	6	8	
Nonintestinal	9	7	
Location			0.576
Cardiac stomach bottom	4	4	
Body	1	1	
Antrum	5	6	
Angle	1	3	
Whole stomach	4	1	
Differentiation			0.682
Well differentiation	3	5	
Poor differentiation	12	10	
*T*			0.190
*T*1	1	1	
*T*2	2	2	
*T*3	7	12	
*T*4	5	0	
*N*			0.963
*N*0	4	4	
*N*1	3	4	
*N*2	4	3	
*N*3	4	4	
Cancer nodules			0.705
0	9	11	
1-2	5	3	
3 and above	1	1	
Vascular invasion			1.000
Yes	8	7	
No	7	8	
Nerve invasion			0.427
Yes	6	3	
No	9	12	
Surgery			0.264
Subtotal	7	11	
Total	8	4	
Liver metastasis			0.175
Yes	4	0	
No	11	15	
Other metastasis			0.651
Yes	2	4	
No	13	11	

## Data Availability

The data used to support the findings of this study are available from the corresponding author upon request.

## References

[1] Bergstrand C. G., Czar B. (1956). Demonstration of a new protein fraction in serum from the human fetus. *Scandinavian Journal of Clinical and Laboratory Investigation*.

[2] Gitlin D., Perricelli A., Gitlin G. M. (1972). Synthesis of -fetoprotein by liver, yolk sac, and gastrointestinal tract of the human conceptus. *Cancer Research*.

[3] Daniele B., Bencivenga A., Megna A. S., Tinessa V. (2004). *α*-fetoprotein and ultrasonography screening for hepatocellular carcinoma. *Gastroenterology*.

[4] O’Conor G. T., Tatarinov Y. S., Abelev G. I., Uriel J. (1970). A collaborative study for the evaluation of a serologic test for primary liver cancer. *Cancer*.

[5] Motoyama T., Watanabe H., Yamamoto T., Sekiguchi M. (1987). Production of alpha-fetoprotein by human germ cell tumors in vivo and in vitro. *Pathology International*.

[6] Yasunami R., Hashimoto Z., Ogura T., Hirao F., Yamamura Y. (1981). Primary lung cancer producing alpha-fetoprotein: a case report. *Cancer*.

[7] Saito S., Hatano T., Hayakawa M., Koyama Y., Ohsawa A., Iwamasa A. T. (1989). Studies on alpha-fetoprotein produced by renal cell carcinoma. *Cancer*.

[8] Liu X., Cheng Y., Sheng W. (2010). Clinicopathologic features and prognostic factors in alpha-fetoprotein-producing gastric cancers: analysis of 104 cases. *Journal of Surgical Oncology*.

[9] Nagai E., Ueyama T., Yao T., Tsuneyoshi M. (1993). Hepatoid adenocarcinoma of the stomach. A clinicopathologic and immunohistochemical analysis. *Cancer*.

[10] Motoyama T., Aizawa K., Watanabe H., Fukase M., Saito K. (1993). *α*-Fetoprotein producing gastric carcinomas: a comparative study of three different subtypes. *Pathology International*.

[11] Alpert E., Pinn V. W., Isselbacher K. J. (1971). Alpha-fetoprotein in a patient with gastric carcinoma metastatic to the liver. *New England Journal of Medicine*.

[12] Wang D., Li C., Xu Y. (2015). Clinicopathological characteristics and prognosis of alpha-fetoprotein positive gastric cancer in Chinese patients. *International Journal of Clinical and Experimental Pathology*.

[13] Sun W., Liu Y., Shou D. (2015). AFP (alpha fetoprotein): who are you in gastrology?. *Cancer Letters*.

[14] Hirajima S. (2013). Liver metastasis is the only independent prognostic factor in AFP-producing gastric cancer. *World Journal of Gastroenterology*.

[15] Kobayashi T. K., Gotoh T., Kamachi M., Watanabe S., Sawaragi I. (1988). Immunocytochemical presentation of alpha-fetoprotein-producing gastric cancer in ascitic fluid: a case study. *Diagnostic Cytopathology*.

[16] Sano M., Inamoto Y., Nagamine N. (1992). Ovarian and hepatic metastases of gastric carcinoma associated with high serum levels of human chorionic gonadotropin (hCG), alpha-fetoprotein (AFP), and carcinoembryonic antigen (CEA): a case report. *Internal Medicine*.

[17] Umekawa Y., Watanabe M., Ikeda S., Fukumoto S., Hirakawa H., Shimada Y. (1994). Alpha-fetoprotein-producing early gastric cancer accompanying liver cirrhosis: a case report. *Journal of Gastroenterology*.

[18] Ishikura H., Kirimoto K., Shamoto M. (1986). Hepatoid adenocarcinomas of the stomach: an analysis of seven cases. *Cancer*.

[19] Kinjo T., Taniguchi H., Kushima R. (2012). Histologic and immunohistochemical analyses of *α*-fetoprotein-producing cancer of the stomach. *The American Journal of Surgical Pathology*.

[20] Metzgeroth G., Ströbel P., Baumbusch T., Reiter A., Hastka J. (2010). Hepatoid adenocarcinoma - review of the literature illustrated by a rare case originating in the peritoneal cavity. *Onkologie*.

[21] Inagawa S., Shimazaki J., Hori M. (2001). Hepatoid adenocarcinoma of the stomach. *Gastric Cancer*.

[22] Kumashiro Y., Yao T., Aishima S. (2007). Hepatoid adenocarcinoma of the stomach: histogenesis and progression in association with intestinal phenotype. *Human Pathology*.

[23] Liu X., Cheng Y., Sheng W. (2010). Analysis of clinicopathologic features and prognostic factors in hepatoid adenocarcinoma of the stomach. *The American Journal of Surgical Pathology*.

[24] Kodama T., Kameya T., Hirota T. (1981). Production of alpha-fetoprotein, normal serum proteins, and human chorionic gonadotropin in stomach cancer: histologic and immunohistochemical analyses of 35 cases. *Cancer*.

[25] Trompetas V., Varsamidakis N., Frangia K., Polimeropoulos V., Kalokairinos E. (2003). Gastric hepatoid adenocarcinoma and familial investigation: does it always produce alpha-fetoprotein?. *European Journal of Gastroenterology & Hepatology*.

[26] Aoyagi K., Koufuji K., Yano S. (2003). Alpha-fetoprotein-producing early gastric cancer: report of two cases. *The Kurume Medical Journal*.

[27] Terada T. (2012). Simultaneous hepatocyte paraffin-1-positive *α*-fetoprotein-producing gastric adenocarcinoma and gastric mucosal-associated lymphoid tissue. *Annals of Diagnostic Pathology*.

[28] Liu X., Sheng W., Wang Y. (2012). An analysis of clinicopathological features and prognosis by comparing hepatoid adenocarcinoma of the stomach with AFP-producing gastric cancer. *Journal of Surgical Oncology*.

[29] Lin H. J., Hsieh Y. H., Fang W. L., Huang K. H., Li A. F. Y. (2014). Clinical manifestations in patients with alpha-fetoprotein-producing gastric cancer. *Current Oncology*.

[30] Inoue M., Sano T., Kuchiba A., Taniguchi H., Fukagawa T., Katai H. (2010). Long-term results of gastrectomy for *α*-fetoprotein-producing gastric cancer. *British Journal of Surgery*.

[31] Chen Y., Qu H., Jian M., Sun G., He Q. (2015). High level of serum AFP is an independent negative prognostic factor in gastric cancer. *The International Journal of Biological Markers*.

